# Circular DNA intermediates in the generation of large human segmental duplications

**DOI:** 10.1186/s12864-020-06998-w

**Published:** 2020-08-26

**Authors:** Javier U. Chicote, Marcos López-Sánchez, Tomàs Marquès-Bonet, José Callizo, Luis A. Pérez-Jurado, Antonio García-España

**Affiliations:** 1 Research Unit, Hospital Universitari de Tarragona Joan XXIII, Institut d’Investigació Sanitària Pere Virgili, Universitat Rovira i Virgili, 43005 Tarragona, Spain; 2 Genetics Unit, Departament de Ciències Experimentals i de la Salut, Universitat Pompeu Fabra, 08003 Barcelona, Spain; 3 Hospital del Mar Research Institute (IMIM) and Centro de Investigación Biomédica en Red de Enfermedades Raras (CIBERER), 08003 Barcelona, Spain; 4 Institut de Biologia Evolutiva (CSIC-UPF), Departament de Ciències Experimentals i de la Salut, Universitat Pompeu Fabra, 08003 Barcelona, Spain; 5 Catalan Institution of Research and Advanced Studies (ICREA), 08010 Barcelona, Spain; 6 CNAG-CRG, Centre for Genomic Regulation, Barcelona Institute of Science and Technology (BIST), 08028 Barcelona, Spain; 7 Department of Ophthalmology, Hospital Universitari de Tarragona Joan XXIII, Institut d’Investigació Sanitària Pere Virgili, Universitat Rovira i Virgili, 43005 Tarragona, Spain; 8 SA Clinical Genetics, Women’s and Children’s Hospital, South Australian Health and Medical Research Institute (SAHMRI) & University of Adelaide, Adelaide, SA 5000 Australia

**Keywords:** Segmental duplications, Circular DNA, Human genome evolution, X-Y transposed region, Chromoanasynthesis,, MMBIR/FoSTeS, NHEJ, Copy number variants

## Abstract

**Background:**

Duplications of large genomic segments provide genetic diversity in genome evolution. Despite their importance, how these duplications are generated remains uncertain, particularly for distant duplicated genomic segments.

**Results:**

Here we provide evidence of the participation of circular DNA intermediates in the single generation of some large human segmental duplications. A specific reversion of sequence order from A-B/C-D to B-A/D-C between duplicated segments and the presence of only microhomologies and short indels at the evolutionary breakpoints suggest a circularization of the donor ancestral locus and an accidental replicative interaction with the acceptor locus.

**Conclusions:**

This novel mechanism of random genomic mutation could explain several distant genomic duplications including some of the ones that took place during recent human evolution.

## Background

Gross genome rearrangements, such as deletions, amplifications, inversions and duplications, are an important source of genetic structural variation for natural selection. Genomic duplications constitute one of the main driving forces for acquiring novel gene functions [1]. Segmental duplications (SDs), which account for over 5% of the human genome, are defined by consensus as duplicated genomic sequences larger than 1-Kb and with an identity over 90% [2–4]. Among humans and great apes, recent SDs provide a substantial fraction of the genetic differences that might underlie the different phenotypes of these species [5, 6]. Additionally, SDs are also susceptibility factors for genomic disorders, a group of human genetic diseases characterized by recurrent genomic rearrangements mediated by non-allelic homologous recombination (NAHR) [7–9]. Understanding the mechanisms involved in SDs’ generation may provide new insights into evolutionary events associated with speciation, adaptation, polymorphic variation, and disease [5, 6, 10]. Proposed mechanisms for the origin of gene duplication include unequal crossing over, retrotransposition, and chromosomal or genome duplication [11]. While unequal crossing over could explain the generation of tandem duplications in proximity on the same chromosome, the generation of interspersed intra-chromosomal and inter-chromosomal duplications is difficult to explain by this mechanism [12].

To our knowledge, circular DNA intermediates generated without classical transposition and independent of homologous recombination have been proposed to mediate genomic duplications in a few eukaryotic organisms. In yeast, where a 16 clusters of five open reading frames have integrated in multiple occasions and in diverse genomic locations in the genome of two industrial strains of *Saccharomyces cerevisiae* [13]; in a basal vertebrate, the Nile tilapia fish, generating a 28 Kb duplication of the vasa gene [14]; and in a single mammal, as the mechanism for two translocations of 492 and 575-kilobases that included the *KIT* gene causing the dominantly inherited color sidedness phenotype in domesticated cattle [15].

In this study we provide evidence for the involvement of replicative circular DNA intermediates in the duplication of sixteen large (> 20-kilobase) genomic segments evolutionarily preserved in the human genome. This novel mechanism of DNA duplication could explain some distant genomic duplications that took place during recent human genomic evolution.

## Results

### Identification of human genomic duplications with an A-B/C-D to B-A/D-C change in sequence order

**Fig. 1:**
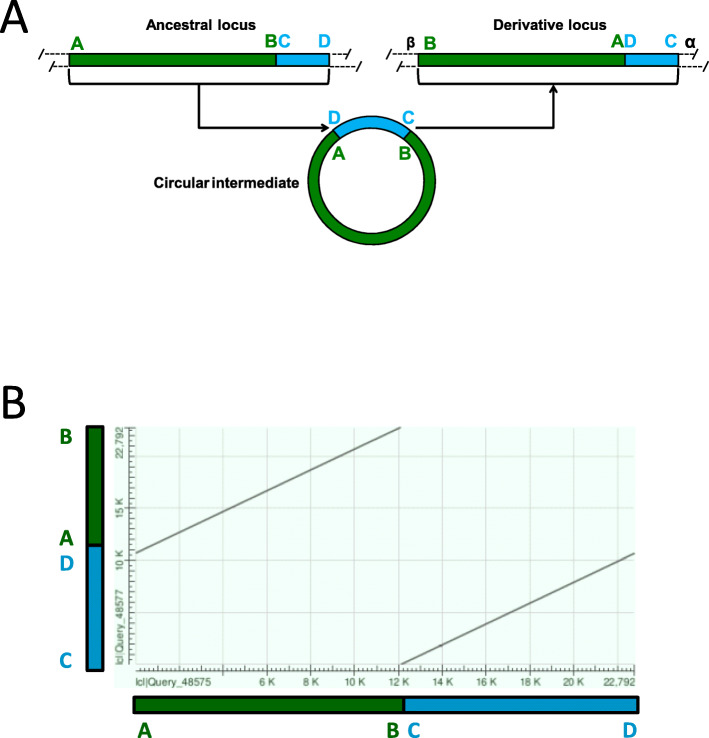
Specific A-B/C-D to B-A/D-C flip in sequence indicative of duplications generated by circular intermediates. **A** Scheme showing the specific change in sequence order in duplications generated via a circular DNA intermediate with unique and distinct closing and opening points. Note that the ends of the ancestral locus A and D will appear joined together inside the derivative duplication A/D. Likewise, the ends of de derivative duplication will appear joined together in the ancestral locus B/C. Duplicated sequences are represented by boxes of the same color: A-B (green boxes) and C-D (blue boxes). **B** Corresponding homology plot of the above duplicated segments showing the specific two parallel identity slant lines produced by the specific flip in block sequence order-

The duplication of a chromosome segment with proximal and distal end points A and D by a circular DNA intermediate that opens in a unique and distinct point (B/C) (Fig. 1A), implies the generation of a derivative segment with a specific change in the segment block order: from A-B/C-D to B-A/D-C [13, 14]. This specific change in the segments block order will generate two parallel identity slant lines in homology plots of the duplicated sequences (Fig. 1B). After an initial unexpected observation of this type of rearrangement in the loci of *UPK3C*, which codes for a highly expressed corneal protein recently characterized by some of us [16, 17], we identified (see methods) four inter-chromosomal and twenty intra-chromosomal pairs of human SD clusters with this specific rearrangement including the X-Y transposed region (SD cluster 6) [18] and the Williams syndrome locus (SD cluster 16) [19, 20] (Table S1and Figure S1). Each duplication block A-B and C-D consists of at least of one annotated SD, more if insertions, deletions and/or inversions have occurred during their evolutionary history (Table S1 and Figure S1). Out of these 24 cluster pairs we have further characterized sixteen (1–12 and 17–20) in which we could differentiate the ancestral/original duplicate from the derivative duplicate; hereafter referred to as circular-DNA-mediated SD Pairs 1–16 (cSDPs 1–16) (Table 1and Table S1).

**Table 1:** Size, distance and evolutionary origin of cSDPs

cSDP	Size Ancestral (Kb)	Distance between SD pairs (Mb)	Closer primate without derivative
cSDP1	107	1,83	Gibbon
cSDP2	131	2,45	Gorilla
cSDP3	244	Inter-chromosomal	Chimpanzee
cSDP4	82	58,48	Orangutan
cSDP5	250	13,12	Orangutan
cSDP6	3918	Inter-chromosomal	Chimpanzee
cSDP7	22	9,40	Chimpanzee
cSDP8	83	51,60	Gorilla
cSDP9	40	1,09	Gorilla
cSDP10	91	1,26	Marmoset
cSDP11	84	8,18	Gibbon
cSDP12	203	Inter-chromosomal	Green monkey
cSDP13	152	Inter-chromosomal	Orangutan
cSDP14	145	0,09	Marmoset
cSDP15	76	45,74	Orangutan
cSDP16	57	2,13	Marmoset

### Characterization, origin and evolutionary timing of cSDPs 1–16

The median length of cSDPs ancestral duplicates is 99 Kb (range 22 to 3918 Kb) and the average distance between duplicates is 16.28 Mb (range from 0.09 to 58.48 Mb) (Table 1). The repetitive element content in cSDPs are similar to the content of their corresponding chromosomes (Table S2). Their evolutionary origin determined by cross species comparison showed that cSDP-3, 6, and 7 are human specific, cSDP-2, 8 and 9 appeared in the common ancestor of humans and chimpanzees, cSDP-4, 5, 13 and 15 in the chimpanzee-gorilla ancestor, cSDP-1 and 11 in the gorilla-orangutan ancestor, cSDP-12 in the gibbons and great apes common ancestor, and cSDP-10, 14 and 16 were of more ancient origin appearing first in the common ancestor of new and old world monkeys (Table 1). In accordance with their evolutionary origin the nucleotide identity between duplication pairs ranges from 98.1–99.4% in human specific cSDP-3 and cSDP-7 to 93.5–93,3% identity in cSDP-12 and cSDP-14 that appeared first in gibbons and green monkeys (Table S1).

### Short indels and/or junctional micro-homologies together with absence of sequence homology characterize the cSDPs breakpoint junctions

**Fig. 2:**
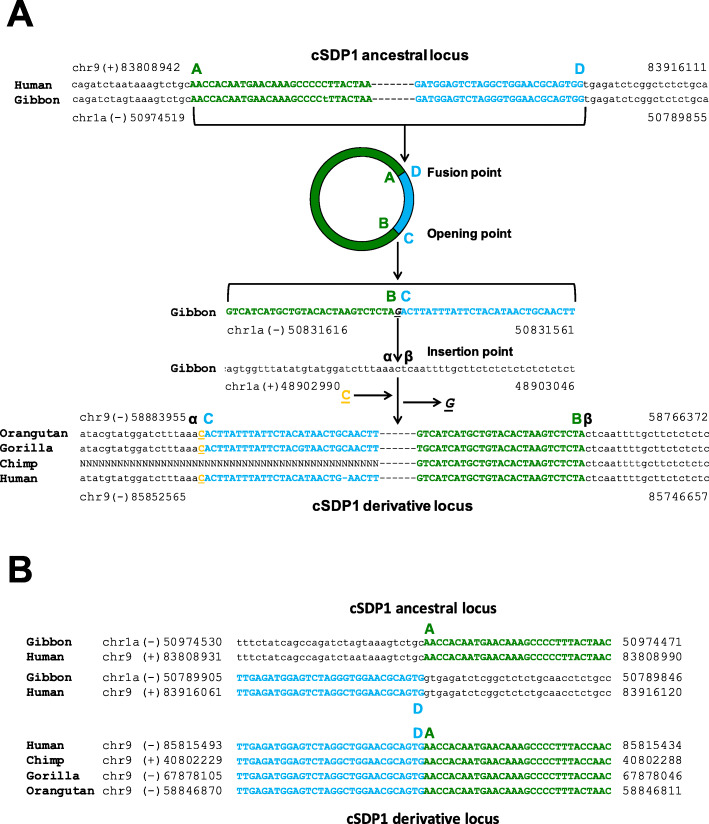
Formation and integration of the circular intermediates, shown as a general example for the generation of cSDP1. **A** Opening and integration of cSDP1 circular intermediate: (Top) ancestral cSDP1 showing sequence fragments flanking the A and D ends of the duplication; (Middle) putative circular intermediate, showing a 56 bp close up of sequence flanking the breaking point, and acceptor sequence in the common ancestor orangutans and gorillas; (Bottom) derivative cSDP1 showing sequence fragments flanking the β-C and B-α junctions. **B** Circular intermediate closing junction. Alignment of the sequences flanking the ends of the ancestral sequence A and D and the AD junction in the derivative sequence. Deleted and inserted base pairs are underlined and shown in italic and orange bold letters respectively. Sequence outside the cSDP is depicted in small letters

**Fig. 3:**
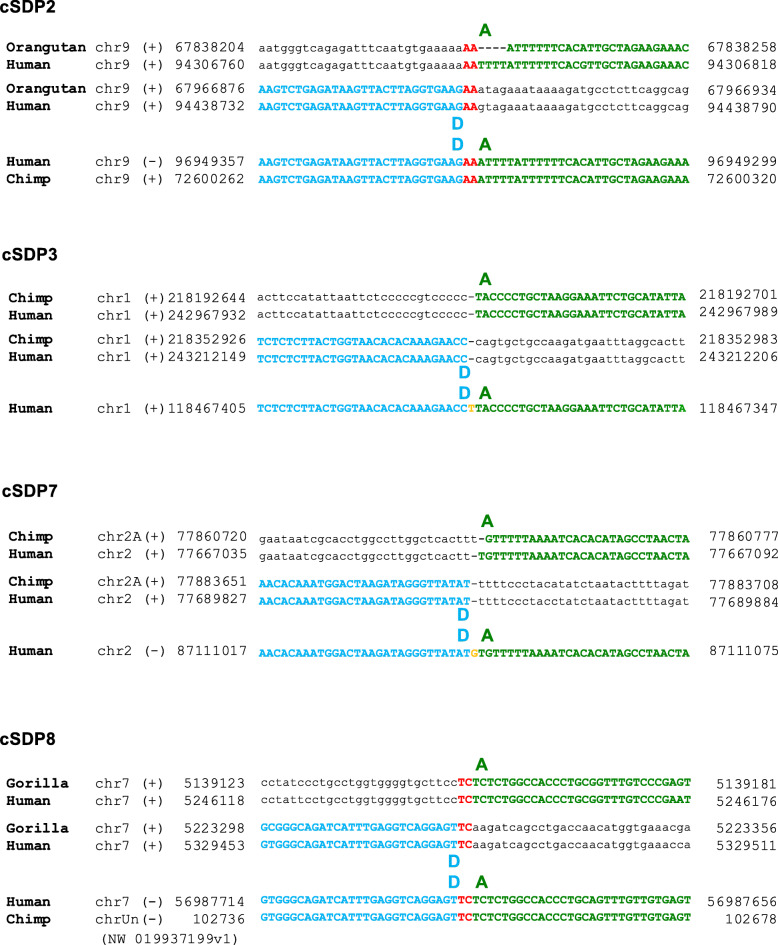
Circular intermediate closing junction of cSDP 2, 3 7 and 8. Alignment of the sequences flanking the ends of the ancestral sequence A and D and the AD junction in the derivative sequence for each duplication. Junction micro-homologies are indicated in red bold letters. Deleted and inserted base pairs are underlined and shown in italic and orange bold letters respectively. Sequence outside the cSDP is depicted in small letters

**Fig. 4:**
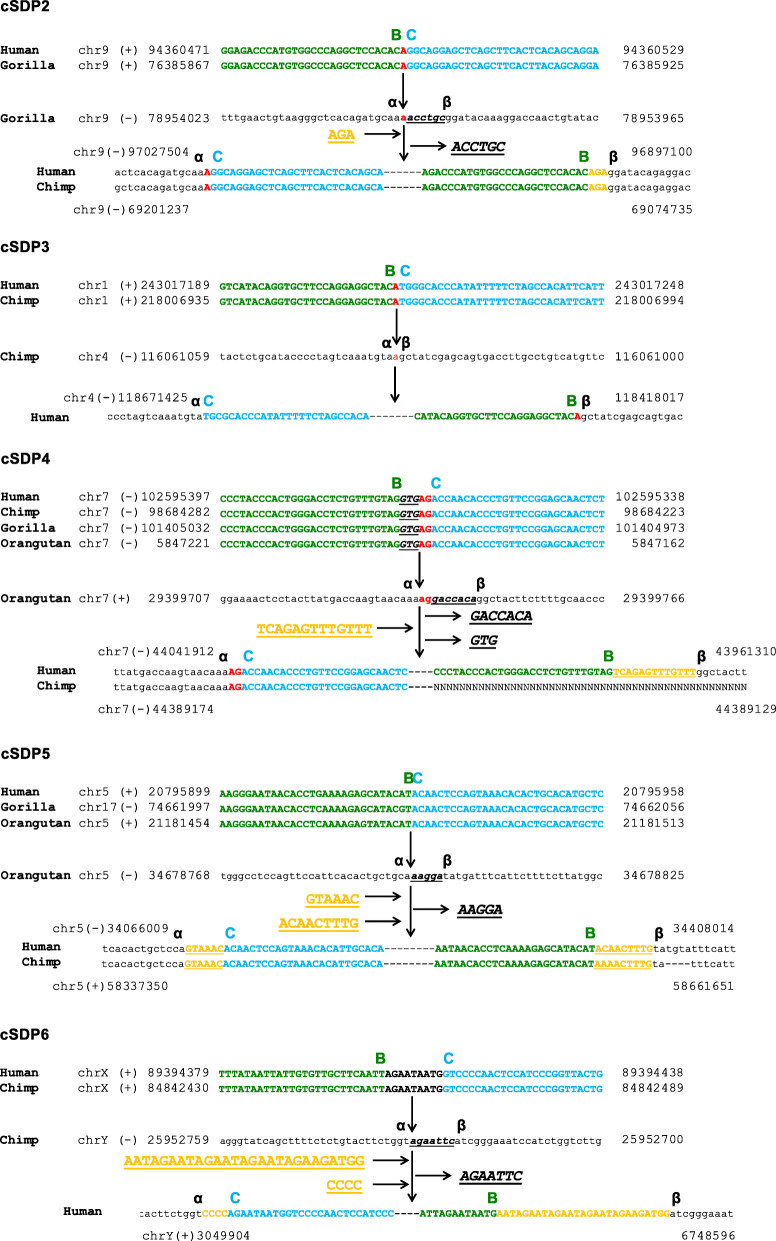
Opening and integration of cSDP 2–6 circular intermediates. For each duplication: (Top) sequence flanking the circular intermediate breaking point; (Middle) acceptor sequence; (Bottom) derivative duplication showing sequence fragments flanking the β-C and B-α junctions. Junction micro-homologies are indicated in red bold letters. Deleted and inserted base pairs are underlined and shown in italic and orange bold letters respectively. Sequence outside the cSDP is depicted in small letters

To analyze how the ancestral donor loci could have circularized and integrated into the derivative acceptor loci, we determined, whenever possible, the exact flanking sequences at the duplication breaking junctions A/D and B/C, and the acceptor sites α/β of the cSDPs (Figs. 2, 3 and 4). We could resolve at the single nucleotide level both the circular intermediate formation (breakpoint A/D) and their insertion (breakpoints B/C and α/β) in three cSDPs (cSDP1, cSDP2 and cSDP3), only the formation in two (cSDP7 and cSDP8) and only the insertion in three (cSDP4 cSDP5 and cSDP6). We could not determine the breakpoints in the remaining eight cSDPs (cSDP9 to cSDP16), due to the presence of other complex SDs, gaps of sequence, or large insertions overlapping the breakpoints in the human and/or in other primate genomes. These analyses showed only gains and/or losses of very short sequences (1 to 27 bp), and/or one or two bp junctional micro-homologies. The fusion of the circular intermediate, (A/D) junction, occurred between two directly adjacent nucleotides in cSDP1, showed one nucleotide insertions in cSDP3 and 7, and junctional micro-homologies of two nucleotides in cSDP2 and cSDP8 (Table 2). The circular intermediate insertion points (breaking junctions B/C and α/β) showed only micro-rearrangements (short indels and microhomologies) (Table 2).

**Table 2:**
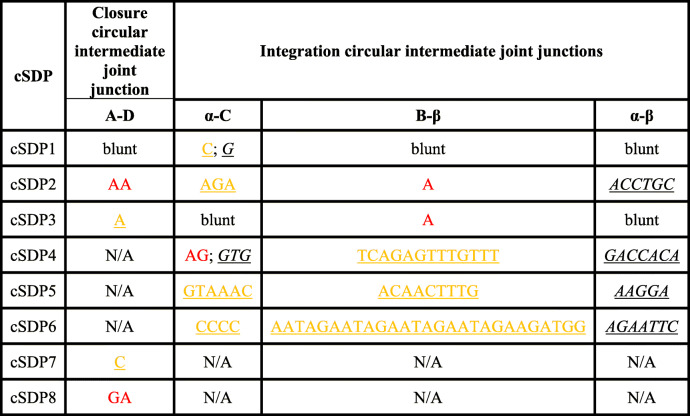
Junctional micro-rearrangements (**homologies/insertions**/***deletions***) generated during the closure and integration of the circular intermediates. Junction micro-homologies are indicated in red letters. Deletions and insertions base pairs are underlined and shown in italic and orange letters respectively

Most evolutionary breakpoints (B/C, α/β, and A and D) mapped to interspersed non-homologous repeat elements, except for the opening point BC in cSDP-3 and cSDP-4, the insertion point α/β in cSDP-1and cSDP2 and the closing points A and D in cSDP-3 (Table S3). Moreover, no significant regions of sequence homology or short inverted repeats were found in the sequences flanking the breaking points (+/− 500 bp) that would allow for the formation of the circular intermediates by either homologous recombination or classical mobilization via a transposon-like element. Also, no direct association of GC content or specific DNA elements including inverted repeats were found at the sequences flanking the duplication breaking points [21].

### Gene content and functional implications

All ancestral duplicates but one (cSDP7) contained genes that resulted in either functional genes, pseudogenes or non-coding genes in the derivative duplication pairs in the cSDPs in which we have resolved at least one breaking point at single nucleotide level. Four ancestral SD blocks contained complete protein-coding genes that generated coding paralogs and five pseudogenes in the derivative copies (Table S4). Two complete copies of core duplicons, expanded human gene families lacking orthologs in other species [5], were found: *NUTM2F* (nuclear testis family member F2) in cSDP-2 and *SPDYE1* (speedy/RINGO cell cycle regulator family member E1) in cSDP-4 (Table S4).

## Discussion

In mammals, the putative involvement of circular intermediates has been only postulated in the generation of two translocations causing a specific phenotype by disruption of the acceptor site in the cattle genome. Whether this was a singular mutation event, a peculiar bovine feature, or a more common mechanism of genome evolution was not determined [15]. We provide evidence of a similar mechanism behind the generation of some large duplications fixed in the human genome.

Our data support the involvement of circular DNA intermediates and suggest a replicative interaction between the donor and acceptor sites in the generation of these duplications. The most parsimonious explanation for the A-B/C-D to B-A/D-C specific flip in sequence order observed between the ancestral and derivative cSDPs would be the circularization of the ancestral cSDP by the fusion of its end points A and D, and the opening of the circular intermediate for re-insertion at single and different breaking points (B/C) (Fig. 1A) [11]. Alternative mechanisms previously suggested, such as transposition followed by inversion that separated the blocks, would place the blocks in inverted direction (B-A/C-D). Thus, a second inversion of exactly the remaining block would be required to generate the observed A-B/C-D to B-A/D-C flips.

Although not specific, additional features that could be related to the generation mechanism of these cSDPs include: (i) the absence of homology in the sequence regions overlapping the breaking junctions of the cSDPs ruling out a homologous recombination mechanism in the formation and in the integration of the circular intermediates; (ii) the presence of micro-rearrangements in the sequences overlapping the breaking junction: short deletions and/or insertions of 1 to-13 bp and/or micro-homologies of 1 or 2 bp; and (iii) a non-tandem location of the ancestral and derivative duplicates. Although the formation and/or insertion of the circular intermediate could only be predicted at the nucleotide level in eight cSDPs, the information provided by the scars left by the circular intermediate formation and integration suggests the implication of a non-replicative non-homologous end joining (NHEJ) mechanism in the formation of the intermediates and is compatible with either NHEJ or to replicative Microhomology-Mediated Break-Induced Replication (MMBIR) / Fork Stalling and Template Switching (FoSTeS) mechanism in its insertion. These informative scars, both in the fusion and insertion breakpoints, are similar to the ones determined in one of the two translocations generated by means of circular intermediates in cattle: a two bp micro-homology typical of NHEJ in the fusion breakpoint of the circular intermediate and micro-duplications and micro-deletions reminiscent of MMBIR in the opening of the intermediate [15]. Furthermore, like in the bovine translocation, the breakpoints of cSDPs mapped to interspersed non-homologous repeat elements suggesting a possible contribution of these elements in the duplication mechanism. On the other hand, the repetitive elements content within ancestral cSDPs matched that of the corresponding chromosomes which suggests repetitive elements within the cSDPs did not contribute to their formation [22].

Three main questions need to be answered: (i) how could a linear segment circularize by fusion of its proximal and distal ends, a requisite for the cSDPs specific flip in sequence, in absence homologous recombination or inverted repeats?; (ii) how could the circular intermediates integrate in the genome in absence of homologous recombination?; and finally (iii), how to account for the large genomic distance between the ancestral and derivative loci?

One possible explanation for the first two questions would be a mechanism like the one reported for chromoanasynthesis [23], localized chromosome rearrangements with variable gains in copy number particularly in cancer genomes. This model postulates that an unexcised interstrand crosslink could lead to breakage of the sister chromatid, with circularization of a retained fragment and integration of the fragment into the genome [23]. In this mechanism, the donor linear segment circularizes by the rejoining of the two ends of the broken chromatid, an event that in our proposed circular intermediate mechanism corresponds to the generation of the fusion point (A/D). Furthermore, this chromatid rejoining will produce the characteristic flip in sequence order observed in the cSDPs. The genome scar signals left by the rejoining of the broken ends A and D in the cSDPs as well as the ones reported in the bovine translocations, two bp micro-homologies, one bp insertions or between two directly adjacent nucleotides suggests a non-replicative mechanism by NHEJ, as previously proposed [15]. Nevertheless, sequence features at the breakpoints are insufficient to distinguish between the NHEJ and MMBIR/FoSTeS mechanisms [24]. In this sense, a replicative MMBIR-like mechanism and homology-directed repair in S-phase has been recently described to explain the formation of circular DNA from the CUP1 locus in yeast [25].

On the other hand, the absence of homology and the presence of only small deletions/insertions as genomic scars and micro-homologies at the integration points of the circular intermediates for cSDPs (breaking junctions B/C and α/β) as found in the bovine translocations suggests the involvement of a replicative MMBIR mechanism [15]. The replicative MMBIR/FoSTeS repair pathways have been implicated in various genomic rearrangements including chromoanasynthesis [23]. In this regard, chromoanasynthesis generated by mutagenesis in *C. elegans* produces two patterns of copy-number increase in the offspring: one pattern with copy number gain from 2 to 3, indicating a simple reintegration of a retained sister chromatid fragment; and a second pattern with up to fivefold copy-number increases of clustered chromosome regions that could be indicative of rolling circle replication mechanism [26, 27]. The copy number pattern of cSDPs of only two suggest the generation of the cSDPs occurred as discrete step by a simple and single reintegration of the recircularized fragment and not by a rolling circle mechanism [28].

The MMBIR/FoSTeS model proposes that after a replication fork stalls the polymerase can switch templates and, depending upon the relative location and orientation of the replication origins, results in directed or inverted tandem duplication, inversion, translocation, or more complex rearrangements [29–31].

Additionally, it has been proposed that, although the involved forks in MMBIR/FoSTeS could be separated by sizeable linear distances or in different chromosomes, they must be adjacent or in close proximity in three-dimensional space, perhaps within replication factories [32]. Further analyses of SDs in human and other species’ as well as in cancer cells and the study of non-recurrent de novo duplications in somatic cells with bioinformatic and experimental tools [4, 33] are needed to define the real role of these circular intermediates in genome plasticity during evolution, health and disease.

## Conclusions

In summary, to our knowledge, this is the first example of novel copy-number-variant-generating mechanism involving an accidental replicative interaction and switching events between the donor and the acceptor locus following uncontrolled replication of a large genomic segment. MMBIR/FoSTeS acting in the germline may produce duplications in the offspring that as in our case could be fixed by natural selection [30]. This novel mechanism of random genomic mutation could explain some of the genomic duplication rearrangements that took place during the recent evolution of the human genomic.

## Methods

### Identification of SD cluster pairs with an A-B/C-D to B-A/D-C change in block order

To visually detect clusters of SDs with the specific flip in sequence from A-B/C-D to B-A/D-C, we scanned all chromosomes using as a template the Chromosomal views (simple) of segmental duplications in the segmental duplications database from UCSC Web site, which depicts SDs > = 1 kb and > = 90% identity site in the hg19 human assembly [2, 34, 35]. Specifically, we look for clusters of SDs that were in the same orientation but with an adjacent inverted order of SD blocks between the two loci. The coordinates of the duplications found with these characteristics were converted to the hg38 assembly, and the duplicated sequences were retrieved and aligned with the NCBI standard nucleotide blast align two sequences tool at default parameters. The alignment results were downloaded as homology plots with the Dot Matrix View of the same Web page.

### Characterization and ancestral origin of SD cluster pairs

For comparative genomics in primates, ancestor identification and prediction of evolutionary rearrangements we used the Blat and Genome convert tools of the UCSC Web site.

Detailed sequence of the cSDPs acceptor sites α/β was determined in the closer primate species (Chimpanzee, assembly panTro6; Corilla, assembly gorGor4; Orangutan, assembly ponAbe3, Gibbon, assembly nomLeu3; Green Monkey, assembly chlSab2; Marmoset assembly calJac3) before the apparition of duplications using the flanking sequences of the derivative cSDPs. The analysis of repetitive elements presence in the duplications breakpoints 500 nucleotide flanking sequences was performed with RepeatMasker program [36] with default parameters at the Web site. Gene content was determined using Gencode release 32 annotation [37] from the UCSC web site.

### Computational detection of SD cluster pairs

To further search undetected cluster pairs in the human genome we created an R algorithm that tested all SDs in hg19 genome build by pairs, searching SD cluster pairs that could constitute the breakpoint B/C (see Supplementary Methods). The first steps in the analysis involved filtering SDs from the genome to obtain a dataset of SDs where to search for compatible SD cluster pairs. These filters removed low-homology (< 0.93) SDs, high density SD regions, high repetitive SD elements (> 4 repetitions), and SDs located in telomeric and centromeric regions. After applying the detection algorithm to the filtered SDs dataset, we extended the detected cluster pairs to include SDs that could constitute the A-B and C-D blocks of the putative cSDP SD cluster pairs. Finally, the resulting regions were visually inspected and checked using the Chromosomal views and plotted with the re-DOT-table software and the Dot Matrix View of the NCBI Web page to remove those regions not compatible with the mechanism and the breakpoint junctions described previously. Out of the 53,000 SDs in the hg19 segmental duplication database and after filtering for SDs with low homology (less than 0,93), for SDs not present in canonical chromosomes, or present in centromeric or complex regions (regions more than 10 SDs) we obtained 6991 unique SDs that when analyzed with the algorithm yielded 160 hits of putative SD clusters pairs (Table S5). Of these 141 where discarded because of unreliable homology plots, absence of defined breaking junctions or lack of correspondence with the hg38 assembly.

## Supplementary information

**Additional file 1: Supplementary Figure S1**. Segmental duplication cluster pairs 1–24 and corresponding homology plots. Segmental duplications included in the duplication clusters (Duplication blocs) retrieved from UCSC Genome Browser snapshots are numbered and highlighted inside green or blue boxes. Specific changes in 5′ to 3′ sequence order are indicated as A-B to B-A, and C-D to D-C or as b-a and d-c when in the complementary strand. Ancestral and derivative cluster copies are represented in the homology plots on the X-axis and Y-axis respectively. Clusters and duplication coordinates are shown in Table S1.

**Additional file 2: Table S1**. Genomic coordinates of duplicated SD clusters and blocks and identity percentage between duplicates. * Duplication 1 in SD clusters 15 and 16 is present twice because there are SD blocks with different sizes between duplication 2 and duplication. **Table S2**. Ratio of repetitive elements content size versus total size of cSDPs or corresponding chromosomes. **Table S3**. Repetitive elements overlapping the closing, opening and insertion breaking junctions during the formation and integration of the circular intermediates. Percentage of repetitive elements in 500 bp of sequence flanking each side of the respective junction are shown in parenthesis and in bold numbers. **Table S4**. NCBI RefSeq curated elements described in the cSDP regions. This table shows the different elements described in the RefSeq curated database that are included in the cSDP regions. In the column “Paralog genes” those genes that may have paralog genes are highlighted in bold. Anc: ancestral; der: derivative; dup: duplicon; BX: Block number X of the cSDP. *: UPK3c is not described in RefSeq, but corresponds to UPK3BL. EST DB249571 shows the expression on the derivative sequence of the first 3 exons of ancestral UPK3c. **Table S5**. Description of the 160 putative SD cluster pairs. This table shows the 160 hits of putative SD clusters pairs, highlighting in green colour those 34 which have a reliable homology plot and there is a breaking junction between the two SD cluster pairs. Dup1: Duplication cluster 1; Dup2: Duplication cluster 2; Alignment length: Length of the alignment between Dup1 and Dup2 in nucleotides; Aligned matches: Number of matching nucleotides in the alignment in nucleotides; Match fraction: Fraction of matching nucleotides; SD blocks: Number of blocks involved in the putative SD cluster pairs; AB: Values belonging to the A-B block described in the proposed mechanism; CD: Values belonging to the C-D block described in the proposed mechanism; Duplication color code: green - putative SD cluster pair with reliable homology plot and a breaking junction between the SD cluster pairs.

**Additional file 3:.** Supplementary methods.

## Data Availability

No new data were generated in this study. The genomes/datasets analyzed in this study can be found at the following links: **panTro6** http://genome-euro.ucsc.edu/cgi-bin/hgTracks?org=Chimp&db=panTro6 **gorGor4** http://genome-euro.ucsc.edu/cgi-bin/hgTracks?db=gorGor4 **ponAbe3** http://genome-euro.ucsc.edu/cgi-bin/hgTracks?org=Orangutan&db=ponAbe3 **nomLeu3** http://genome-euro.ucsc.edu/cgi-bin/hgTracks?db=nomLeu3 c**hlSab2** http://genome-euro.ucsc.edu/cgi-bin/hgTracks?db=chlSab2 **calJac3** http://genome-euro.ucsc.edu/cgi-bin/hgTracks?db=calJac3 **hg19** http://genome-euro.ucsc.edu/cgi-bin/hgTracks?db=hg19 **hg38** http://genome-euro.ucsc.edu/cgi-bin/hgTracks?db=hg38 **Segmental.Duplications.hg19** http://hgdownload.soe.ucsc.edu/goldenPath/hg19/database/genomicSuperDups.txt.gz **Segmental.Duplications.hg38.** http://hgdownload.soe.ucsc.edu/goldenPath/hg38/database/genomicSuperDups.txt.gz **Bsgenome.Hsapiens.UCSC.hg19.** https://bioconductor.org/packages/release/data/annotation/html/BSgenome.Hsapiens.UCSC.hg19.html **Bsgenome.Hsapiens.UCSC.hg38.** https://bioconductor.org/packages/release/data/annotation/html/BSgenome.Hsapiens.UCSC.hg38.html

## Abbreviations

cSDPs: Circular-DNA-mediated SD Pairs
FoSTeS: Fork Stalling and Template Switching
Kb: Kilo base pairs
MMBIR: Microhomology-Mediated Break-Induced Replication
NAHR: Non-allelic homologous recombination
NHEJ: Non-homologous end joining
SDs: Segmental duplications

## References

[1] Ohno S (1970). Evolution by gene duplication.

[2] Eichler EE (2001). Recent duplication, domain accretion and the dynamic mutation of the human genome. Trends Genet.

[3] Bailey JA, Yavor AM, Massa HF, Trask BJ, Eichler EE (2001). Segmental duplications: organization and impact within the current human genome project assembly. Genome Res.

[4] Pu L, Lin Y, Pevzner PA (2018). Detection and analysis of ancient segmental duplications in mammalian genomes. Genome Res.

[5] Marques-Bonet T, Eichler EE (2009). The evolution of human segmental duplications and the core duplicon hypothesis. Cold Spring Harb Symp Quant Biol.

[6] Dennis MY, Eichler EE (2016). Human adaptation and evolution by segmental duplication. Curr Opin Genet Dev.

[7] Emanuel BS, Shaikh TH (2001). Segmental duplications: an 'expanding' role in genomic instability and disease. Nat Rev Genet.

[8] Carvalho CM, Zhang F, Lupski JR (2010). Genomic disorders: a window into human gene and genome evolution. Proc Natl Acad Sci.

[9] Stankiewicz P, Lupski JR (2002). Molecular-evolutionary mechanisms for genomic disorders. Curr Opin Genet Dev.

[10] Jiang Z, Tang H, Ventura M, Cardone MF, Marques-Bonet T, She X, Pevzner PA, Eichler EE (2007). Ancestral reconstruction of segmental duplications reveals punctuated cores of human genome evolution. Nat Genet.

[11] Mendivil Ramos O, Ferrier DE (2012). Mechanisms of gene duplication and translocation and Progress towards understanding their relative contributions to animal genome evolution. Int J Evol Biol.

[12] Reams AB, Roth JR. Mechanisms of gene duplication and amplification. Cold Spring Harb Perspect Biol. 2015;7:a016592. 10.1101/cshperspect.a016592 PMC4315931 25646380

[13] Borneman AR, Desany BA, Riches D, Affourtit JP, Forgan AH, Pretorius IS, Egholm M, Chambers PJ (2011). Whole-genome comparison reveals novel genetic elements that characterize the genome of industrial strains of Saccharomyces cerevisiae. PLoS Genet.

[14] Fujimura K, Conte MA, Kocher TD (2011). Circular DNA intermediate in the duplication of Nile tilapia vasa genes. PLoS One.

[15] Durkin K, Coppieters W, Drogemuller C, Ahariz N, Cambisano N, Druet T, Fasquelle C, Haile A, Horin P, Huang L (2012). Serial translocation by means of circular intermediates underlies colour sidedness in cattle. Nature.

[16] Desalle R, Chicote JU, Sun TT, Garcia-Espana A (2014). Generation of divergent uroplakin tetraspanins and their partners during vertebrate evolution: identification of novel uroplakins. BMC Evol Biol.

[17] Chicote JU, DeSalle R, Segarra J, Sun TT, Garcia-Espana A (2017). The Tetraspanin-associated Uroplakins family (UPK2/3) is evolutionarily related to PTPRQ, a Phosphotyrosine phosphatase receptor. PLoS One.

[18] Schwartz A, Chan DC, Brown LG, Alagappan R, Pettay D, Disteche C, McGillivray B, de la Chapelle A, Page DC (1998). Reconstructing hominid Y evolution: X-homologous block, created by X-Y transposition, was disrupted by Yp inversion through LINE-LINE recombination. Hum Mol Genet.

[19] Antonell A, de Luis O, Domingo-Roura X, Perez-Jurado LA (2005). Evolutionary mechanisms shaping the genomic structure of the Williams-Beuren syndrome chromosomal region at human 7q11.23. Genome Res.

[20] Dennis MY, Harshman L, Nelson BJ, Penn O, Cantsilieris S, Huddleston J, Antonacci F, Penewit K, Denman L, Raja A (2017). The evolution and population diversity of human-specific segmental duplications. Nat Ecol Evol.

[21] Carvalho CM, Ramocki MB, Pehlivan D, Franco LM, Gonzaga-Jauregui C, Fang P, McCall A, Pivnick EK, Hines-Dowell S, Seaver LH (2011). Inverted genomic segments and complex triplication rearrangements are mediated by inverted repeats in the human genome. Nat Genet.

[22] Møller HD, Ramos-Madrigal J, Prada-Luengo I, Gilbert MTP, Regenberg B (2020). Near-random distribution of chromosome-derived circular DNA in the condensed genome of pigeons and the larger, More Repeat-Rich Human Genome. Genome Biol Evol.

[23] Willis NA, Rass E, Scully R (2015). Deciphering the code of the Cancer genome: mechanisms of chromosome rearrangement. Trends Cancer.

[24] Yang L, Luquette LJ, Gehlenborg N, Xi R, Haseley PS, Hsieh CH, Zhang C, Ren X, Protopopov A, Chin L, et al. Diverse mechanisms of somatic structural variations in human cancer genomes. Cell. 2013;153:919–29. 10.1016/j.cell.2013.04.010 PMC3704973 23663786

[25] Hull RM, King M, Pizza G, Krueger F, Vergara X, Houseley J (2019). Transcription-induced formation of extrachromosomal DNA during yeast ageing. PLoS Biol.

[26] Meier B, Cooke SL, Weiss J, Bailly AP, Alexandrov LB, Marshall J, Raine K, Maddison M, Anderson E, Stratton MR (2014). C. elegans whole-genome sequencing reveals mutational signatures related to carcinogens and DNA repair deficiency. Genome Res.

[27] Thierry A, Khanna V, Creno S, Lafontaine I, Ma L, Bouchier C, Dujon B. Macrotene chromosomes provide insights to a new mechanism of high-order gene amplification in eukaryotes. Nat Commun. 2015;6:6154. 10.1038/ncomms7154 PMC4317496 25635677

[28] Deans AJ, West SC (2011). DNA interstrand crosslink repair and cancer. Nat Rev Cancer.

[29] Hastings PJ, Ira G, Lupski JR (2009). A microhomology-mediated break-induced replication model for the origin of human copy number variation. PLoS Genet.

[30] Hastings PJ, Lupski JR, Rosenberg SM, Ira G (2009). Mechanisms of change in gene copy number. Nat Rev Genet.

[31] Zhang L, Lu HH, Chung WY, Yang J, Li WH (2005). Patterns of segmental duplication in the human genome. Mol Biol Evol.

[32] Kitamura E, Blow JJ, Tanaka TU (2006). Live-cell imaging reveals replication of individual replicons in eukaryotic replication factories. Cell.

[33] Shao M, Lin Y, Moret B. Sorting genomes with rearrangements and segmental duplications through trajectory graphs. BMC Bioinformatics. 14(Suppl 15):S9. 10.1186/1471-2105-14-S15-S9 PMC3851842 24564345

[34] Bailey JA, Gu Z, Clark RA, Reinert K, Samonte RV, Schwartz S, Adams MD, Myers EW, Li PW, Eichler EE (2002). Recent segmental duplications in the human genome. Science.

[35] She X, Jiang Z, Clark RA, Liu G, Cheng Z, Tuzun E, Church DM, Sutton G, Halpern AL, Eichler EE (2004). Shotgun sequence assembly and recent segmental duplications within the human genome. Nature.

[36] Smit, AFA, Hubley, R & Green, P. RepeatMasker Open-4.0. 2013-2015 <http://www.repeatmasker.org>.

[37] Frankish A, Diekhans M, Ferreira AM (2019). GENCODE reference annotation for the human and mouse genomes. Nucleic Acids Res.

