# Supplementary material for: Circular DNA intermediates in the generation of large human segmental duplications
Source: BMC Genomics. 2020 Aug 26;21:593. doi: 10.1186/s12864-020-06998-w (PMC7450558; doi:10.1186/s12864-020-06998-w)
Supplement: Supplementary file 1 — Additional file 1: Supplementary Figure S1. Segmental duplication cluster pairs 1–24 and corresponding homology plots. Segmental duplications included in the duplication clusters (Duplication blocs) retrieved from UCSC Genome Browser snapshots are numbered and highlighted inside green or blue boxes. Specific changes in 5′ to 3′ sequence order are indicated as A-B to B-A, and C-D to D-C or as b-a and d-c when in the complementary strand. Ancestral and derivative cluster copies are represented in the homology plots on the X-axis and Y-axis respectively. Clusters and duplication coordinates are shown in Table S1. [file 12864_2020_6998_MOESM1_ESM.ppt]

## Slide 1
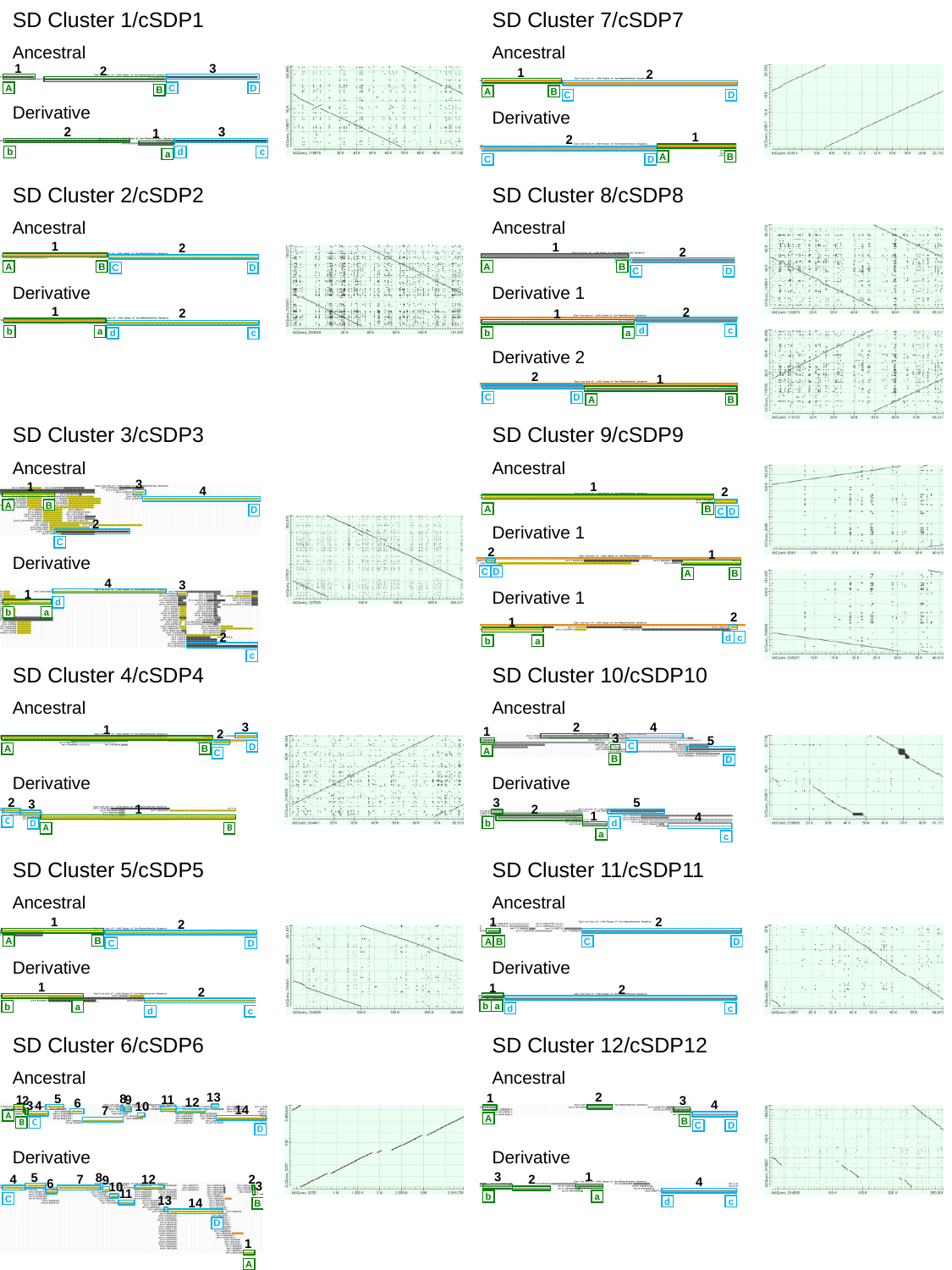

SD Cluster 1/cSDP1
SD Cluster 7/cSDP7
Ancestral
Ancestral
Derivative
Derivative
SD Cluster 2/cSDP2
SD Cluster 8/cSDP8
Ancestral
Ancestral
Derivative
Derivative 1
Derivative 2
SD Cluster 3/cSDP3
SD Cluster 9/cSDP9
Ancestral
Ancestral
Derivative 1
Derivative
Derivative 1
SD Cluster 4/cSDP4
SD Cluster 10/cSDP10
Ancestral
Ancestral
Derivative
Derivative
SD Cluster 5/cSDP5
SD Cluster 11/cSDP11
Ancestral
Ancestral
Derivative
Derivative
SD Cluster 6/cSDP6
SD Cluster 12/cSDP12
Ancestral
Ancestral
Derivative
Derivative

## Slide 2
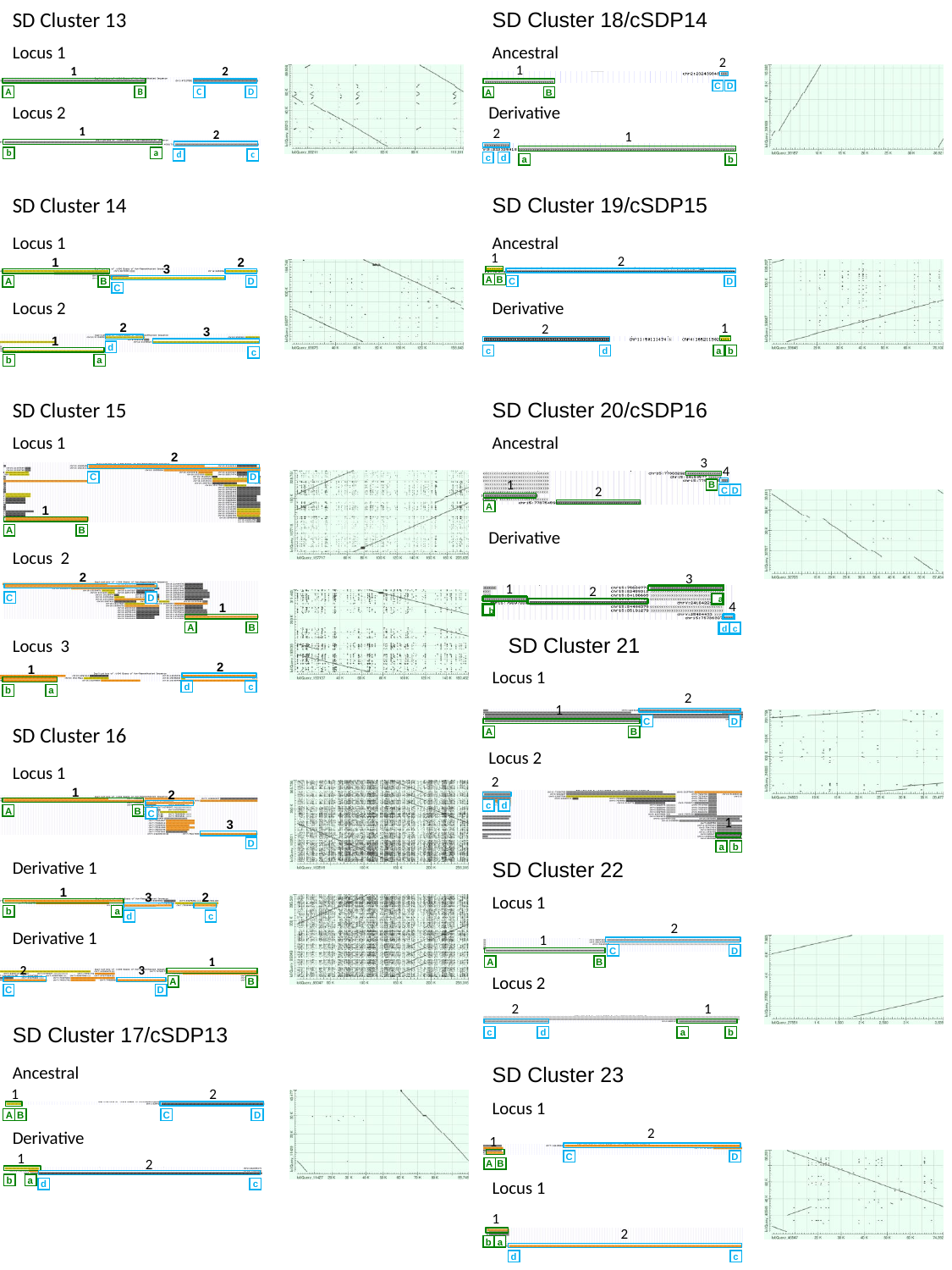

SD Cluster 13
SD Cluster 18/cSDP14
Locus 1
Ancestral
Locus 2
Derivative
SD Cluster 14
SD Cluster 19/cSDP15
Locus 1
Ancestral
Locus 2
Derivative
SD Cluster 15
SD Cluster 20/cSDP16
Locus 1
Ancestral
Derivative
Locus 2
3
1
2
a
4
b
d
c
SD Cluster 21
Locus 3
Locus 1
SD Cluster 16
Locus 2
Locus 1
Derivative 1
SD Cluster 22
Locus 1
Derivative 1
Locus 2
SD Cluster 17/cSDP13
Ancestral
SD Cluster 23
Locus 1
Derivative
Locus 1

## Slide 3
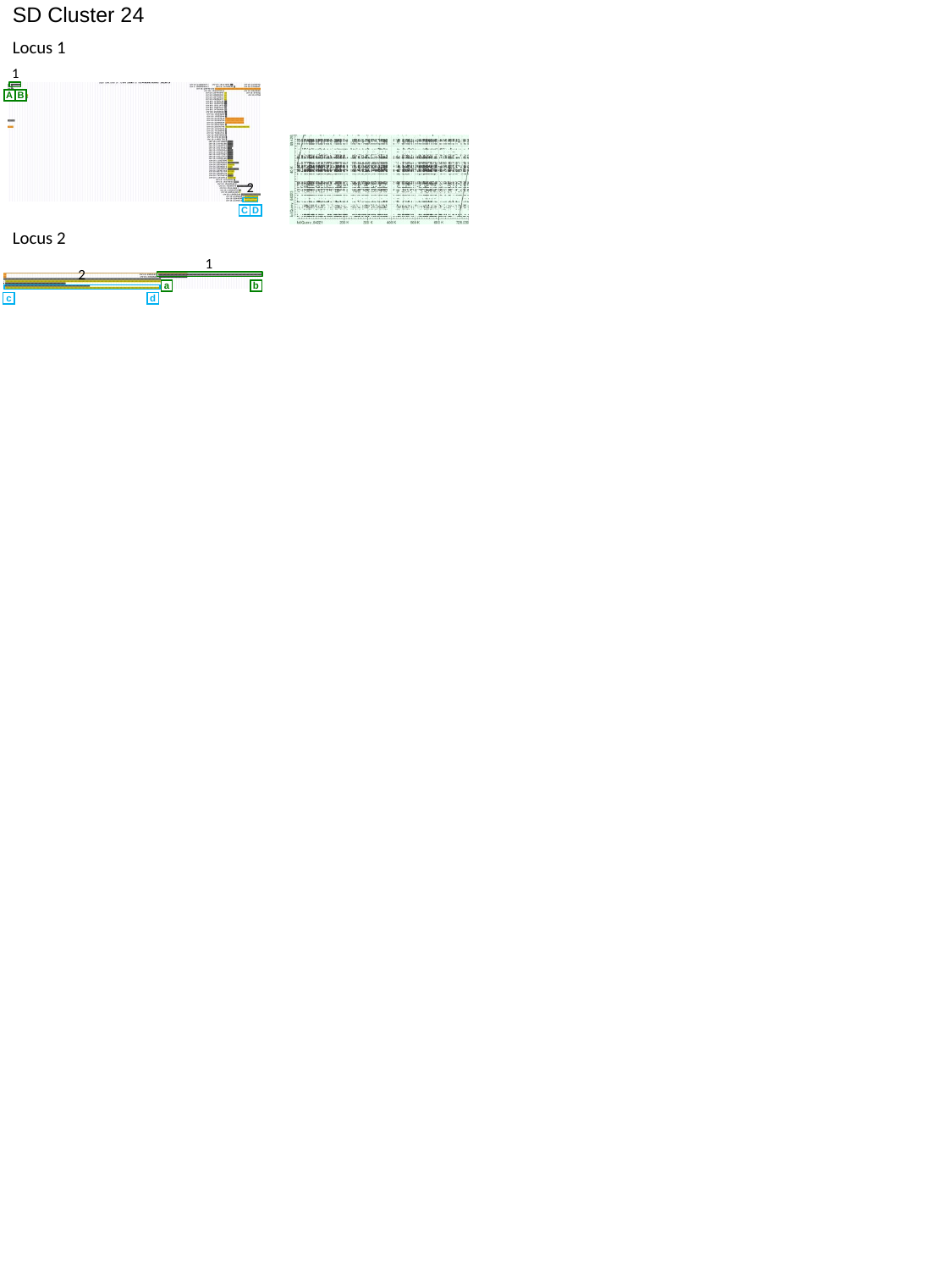

SD Cluster 24
Locus 1
Locus 2
