# Supplementary material for: Circular DNA intermediates in the generation of large human segmental duplications
Source: BMC Genomics. 2020 Aug 26;21:593. doi: 10.1186/s12864-020-06998-w (PMC7450558; doi:10.1186/s12864-020-06998-w)
Supplement: Supplementary file 3 — Additional file 3:. Supplementary methods. [file 12864_2020_6998_MOESM3_ESM.docx]

# Supplementary methods

## Quantitative assessment of SD clusters in the human genomewith an A-B/C-D to B-A/D-C change in block order.

The search for SD cluster was performed using segmental duplications (SDs) tracks downloaded from UCSC webpage for human genome builds hg19 and hg38. In these files, each SD track entry describes a SD, with the chromosome, position, relative orientation, homology and alignment statistics of the duplication blocks involved in the SD. All the analysis was performed in R, using the packages *Rtracklayer* and *data.table* to read the files, *GenomicRanges* to store the SDs in this format, *GenomicAlignments* to store and manipulate genomic alignments, and D3GB to retrieve the centromeric and telomeric positions of the chromosomes. The R packages *BSgenome.Hsapiens.UCSC.hg19*, *BSgenome.Hsapiens.UCSC.hg39*, *Biostrings* and *seqinr* packages were used further in the analysis to retrieve the genomic sequence of the events and check the potential SD cluster pairs .

## Identification of SD duplication blocks

The first steps in the search for SD cluster pairs is the identification of SD duplication blocks that could be involved in a dupliation event to calculate the expected occurrence of events in the human genome. These SD duplication blocks must meet the following conditions that were applied as filters to all reported SDs in the files: 1) Removing repetitive and mobile repetitive elements, because these elements have other mechanisms not related to the mechanisms proposed in this paper to travel and replicate around the genome; 2) Removing SDs with low homology (homology <= 93%) to focus the study in events that can be traced through the primate evolution; 3) Removing SDs located in heterochromatic or centromeric regions, to remove other repetitive and shared elements not removed with the previous filters; and 4) Removing SDs in regions with a density of SDs higher than 10 (Coverage_SDs_ >=10 ) present in high density SD clusters or hotspots of recombination that are challenging to study.

## Scanning for potential SD cluster pairs events

After identifying the compatibles SD duplication blocks, these were scanned in search for one or more SD duplication blocks that could constitute the division between B and C of the SD cluster event in Duplication 1 (Figure 1). These block pairs are identified as tmpAB and tmpCD, its respective reciprocal SDs are identified as otherAB and otherCD and located in Duplication 2 (Figure 1), and all of them must met the following conditions in order to be considered as potential SD cluster events:


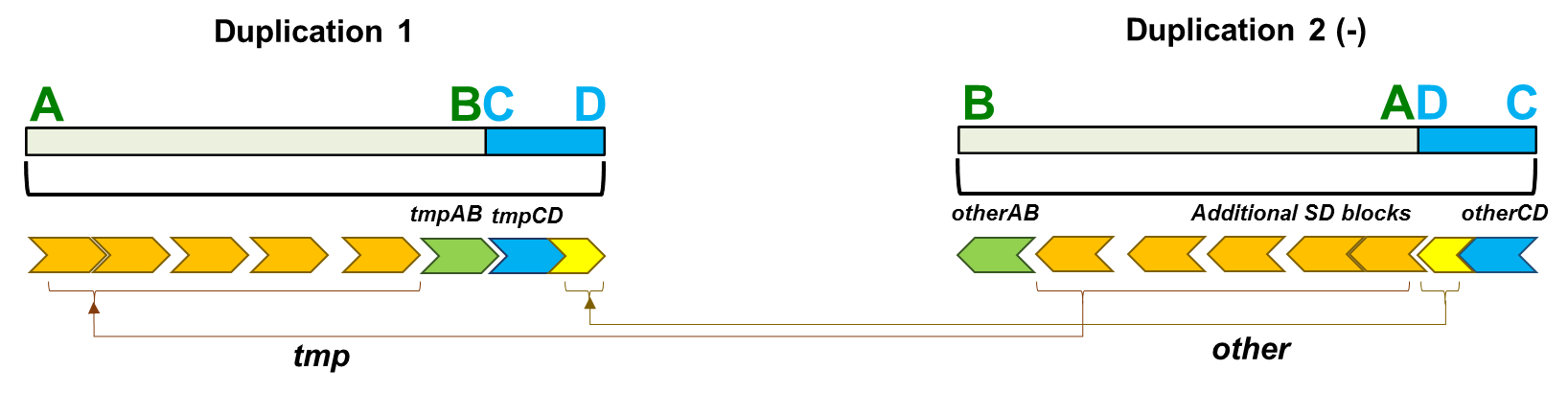


Figure 1: Scanning for potential SD cluster pairs events and extension of the potential events detected. This figure describes the process of scanning for potential events by selecting a SD duplication block in Duplication 1 (tmpAB in green, with its reciprocal block otherAB also in green colour in Duplication 2) and searching for a consecutive block (tmpCD in blue) compatible with the SD cluster event, in this case, in the BADC (-) configuration of Duplication 2. After this scanning step, an extension step is performed where all the segmental duplication blocks in Duplication 2 between otherAB (green) and otherCD (blue) are checked if are compatible with the configuration in Duplication 1. All the compatible blocks (orange and yellow) will be included in the Duplication 1 (tmp) region.

1. Both SD duplication blocks in duplication 1 (tmpAB and tmpCD) and its reciprocal SD duplication blocks in duplication 2 (otherAB and otherCD) are located in the same chromosome (1), with the same alignment orientation (2) and with a maximum overlap in their positions of 20 nt (3). The first two conditions are basic conditions that must be met between the events, but the third condition was established in order to avoid overlapping SD blocks and to include possible small deletions of the breakpoint B-C or fusions that have been observed in the SD cluster pairs preliminary results.

$$\left( 1 \right){Chromosome}_{tmpAB}={Chromosome}_{tmpCD};$$

$${Chromosome}_{otherAB}={Chromosome}_{otherCD}$$

$$\left( 2 \right){Alignment}_{tmpAB-otherAB}={Alignment}_{tmpCD-otherCD}$$

$$\left( 3 \right){Overlap}_{tmpAB-tmpCD}\leq20nt;{Overlap}_{otherAB-otherCD}\leq20nt$$

2. The difference between both SD block pairs homology (tmpAB-otherAB and tmpCD-otherCD) is lower than 0.008 (4). This value was obtained by measuring the mean difference between homology in the SD cluster pairs reported in the preliminary results and was set as a threshold measure of difference between SD blocks.

$$\left( 4 \right)\left| \left( {Hom}_{tmpAB-otherAB}-{Hom}_{tmpCD-otherCD} \right) \right|\leq0.008$$

3. There is a rearrangement in the block’s order position compatible with the SD cluster pairs insertion mechanism, depending on the alignment orientation between duplication 1 and duplication 2 (5).

$$\left( 5 \right){Alignment}_{tmpAB-otherAB}\left\{ \begin{aligned} if+:{End}_{tmpAB}<{End}_{tmpCD};{End}_{otherAB}<{End}_{otherCD} \\ if-:{End}_{tmpAB}<{End}_{tmpCD};{End}_{otherAB}>{End}_{otherCD} \end{aligned} \right.$$

4. The length of the duplication 2 is lower than 12 times the width of the duplication 1 event. This threshold was set considering the largest size difference detected in the preliminary results, from the X transposed event (SD cluster 7). In SD cluster 7, the SD blocks that constitute the B end and the C end of the event have a length between SD ends of 358,891 bp in chromosome X (ancestral, GRCh38) and a size of 3,698,641 in chromosome Y (derivative, GRCh38), resulting in a 10.3 times increase in length of the derivative than ancestral events. This increment was rounded to 12 to include possible larger events (6).

$$\left( 6 \right){Alignment}_{tmpAB-otherAB}\left\{ \begin{aligned} if+:{Length}_{tmpAB-tmpCD}\leq12\cdot{Length}_{otherCD-otherAB} \\ if-:{Length}_{tmpAB-tmpCD}\leq12\cdot{Length}_{otherAB-otherCD} \end{aligned} \right.$$

5. If there is more than one potential event detected for the scanned SD duplication block, we keep only one of them. The compatible SD block is selected accounting first for the least difference in homology (7), and if there are still more than one SD block compatible, keeping the SD with least separation between SD blocks (8).

$$\left( 7 \right)Select{SD}_{CD}wheremin\left[ \left| \left( {Homology}_{tmpAB-otherAB}-{Homology}_{tmpCD-otherCD} \right) \right| \right]$$

$$\left( 8 \right)Select{SD}_{CD}where{Alignment}_{tmpAB-otherAB}\left\{ \begin{aligned} if+:min\left[ {End}_{otherAB}-{End}_{otherCD} \right] \\ if-:min\left[ {End}_{otherCD}-{End}_{otherAB} \right] \end{aligned} \right.$$

## Extending the SD cluster blocks

After detecting the two SD duplication blocks belonging to the breakpoints B and C, we must extend the SD cluster blocks with compatible SDs included in the reciprocal region between B and C breakpoints to add all the segmental duplications involved in the duplication event. This step is performed by scanning all the potential events previously detected and keeping all those SDs that match the following criteria: 1) have the same orientation, 2) Homology difference between the blocks previously reported inferior to 0.005 and3) Non-overlapping SDs with the previously reported blocks.

Once the SD cluster blocks are extended, we performed a filtering step in order to remove duplicated or overlapping events that have arisen due to the SD cluster blocks extension.

## Visual inspection of the detected events

After all the analysis procedure, all the potential events were plotted using the *re-DOT-able* software (<https://www.bioinformatics.babraham.ac.uk/projects/redotable/>) and the blastn suite (<https://blast.ncbi.nlm.nih.gov/Blast.cgi>) to perform a dot-plot visualization of the alignment. In addition, the events are visually inspected in UCSC genome browser for the appropriate genome build used in the search, and sequences were extracted from the *BSgenome.Hsapiens.UCSC.hg19* or *BSgenome.Hsapiens.UCSC.hg38*. Those events without clear insertion points between the A-D or B-C boundaries or with large separation between blocks have been discarded.

## Determination of the GC content in the flanking regions of the SD pairs

GC content of the flanking regions was determined first retrieving the DNA flanking sequences of the SD pairs from the *Bsgenome.Hsapiens.UCSC.hg38* R package. GC content was determined for each DNA flanking sequence using the *GC* function of the *seqinr* R package.

## Repetitive elements proportion in SD cluster pairs

To check if there are differences between the proportion of repetitive elements in SD cluster pairs and the proportion of repetitive elements in the genome, all repetitive elements were retrieved from UCSC RepeatMasker annotation file for hg38 genome build. Taking in consideration only those repetitive elements included in the SD cluster block, the repetitive elements lengths were added up and then divided by the SD cluster block length for each SD cluster pair. The same procedure was done for each chromosome to calculate the repetitive elements ratio, dividing by the chromosome length retrieved from the *BSgenome.Hsapiens.UCSC.hg38 R package*.
